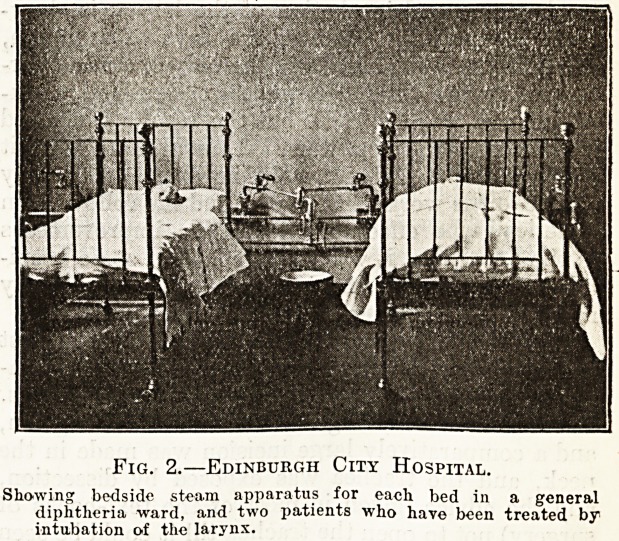# Some Fever Hospitals and Their Work

**Published:** 1912-08-31

**Authors:** A. Knyvett Gordon

**Affiliations:** formerly Medical Superintendent of Monsall Hospital and Lecturer on Infectious Diseases in the University of Machester


### SOME FEVER HOSPITALS AND THEIR WORK.*

### VI.—A Summary of Recent Improvements.

By A. KNYVETT GORDON, M.B. Cantab., formerly Medical Superintendent of Monsall Hospital and Lecturer on Infectious Diseases in the University of Manchester.

In the preceding articles I have taken scarlet fever as a type of infectious disease, and have described some of the methods in use at modern fever hospitals for dealing with the patients so that the risk of cross-infection may be reduced to a minimum. Before leaving this disease it may be as well to summarise the changes that have

Fig. 1.—Leeds City Hospital.

Part of one of the diphtheria wards, showing laryngeal operating-room adjoining, and bedside steam apparatus.

taken place in this respect during the last fifteen years or so.

"Temporary" wooden buildings, which could not be adequately disinfected on account of the porous nature of their walls and floors, have largely been replaced by permanent pavilions constructed of impervious and sterilisable materials. Most authorities have also increased their accommodation so that each patient receives a larger amount of air and floor space than formerly. The overcrowding that often existed, especially during the prevalence of an epidemic, has been shown to be a fruitful source both of cross-infection, and—what is practically the same thing—of so-called septic "complications" also.

Whereas formerly all patients suffering from scarlet fever were treated in the same wards, and without any very great attempt at asepsis on the part of the nurses, most superintendents now segregate the septic cases (either by the barrier system or one of its modifications) in the general wards, or isolate them altogether in side or isolation wards or in a separate building when available.

A very great change has taken place in the development of asepsis in nursing, particularly in the attention paid to the sterilisation of instruments and utensils. *Pari passu* with this, the standard of fever nursing generally, and also with regard to the selection and instruction of nurses, has been considerably raised. Nurses are now generally engaged for a definite period of service, and certificates are awarded at its conclusion after the candidates have also passed examinations in theoretical knowledge.

Improvements have also taken place in the medical staff. The existence of a hospital of any size without a resident medical officer is becoming obsolete, and the bacteriological work, which is so essential to successful treatment, is more generally carried out at the hospital itself, instead of being sent to an outside laboratory, a procedure which often entails dangerous delay.

There is a growing tendency to enlarge the scope of the fever hospital as regards the variety of diseases admitted. In order that this may be done without risk of cross-infection, improvements have taken place in the construction and administration of isolation wards, the most notable of these being the adoption of the partial or complete cubicle systems and the organisation of aseptic technique without structural separation, which has resulted in the method of "bed isolation."

Attempts have been made to reduce the incidence of return cases by decanting the patients from one ward to another before their discharge.

The practice of treating certain cases in the open air has grown in favour, and appears likely to be more generally adopted in the future.

Considerable improvements have taken place in the ambulance services, each patient being nowadays removed separately in charge of a nurse; motor ambulances are being more widely adopted.

### Diphtheria and its Treatment.

We come now to some details connected with the treatment of diphtheria. It should nowadays

Fig. 2.—Edinburgh City Hospital.

Showing bedside steam apparatus for each bed in a general diphtheria ward, and two patients who have been treated by intubation of the larynx.

be hardly necessary to emphasise the importance of administering antitoxic serum at the very earliest period of the disease, and in the fever hospitals themselves this is invariably done, the only difference between them being in regard to the average

* Previous articles appeared on July 13, 20, Aug. 10, 17, 24.

dose of serum that is usually given; in this respect the large doses of 20,000 units and more that used to be in vogue ten years or so ago have given place to a moderate amount of 4,000 to 6,000 units.

It is much to be wished, however, that the custom of administering serum before the admission of the patient to hospital were more common. Notwithstanding the fact that almost all authorities distribute the antitoxin freely and gratuitously to the medical profession for this purpose, the records of almost all of the larger hospitals still show a large percentage of patients admitted on the fourth and later days of disease who have not been thus treated. It has now been proved up to the hilt by repeated and extensive reports, notably those issued by the Metropolitan Asylums Board in London, that the mortality amongst patients who have received serum on the first day of disease is practically nil, and increases rapidly with each day of delay. I have before me as illustrating this point the figures for the City of Edinburgh for 1911, which are as follows:—

| Day of Administration of Serum. | Death-rate per cent. |
|---|---|
| First | .0 |
| Second | 4.0 |
| Third | 9.4 |
| Fourth | 9.8 |
| Fifth, sixth, and seventh | 21.2 |
| After seventh | 27.0 |

While the death-rate of patients admitted to the City Hospital was 11 per cent. as against 28.5 per cent. amongst those treated at home.

Another effect of serum is considerably to reduce the incidence of extension of the disease to the larynx, so that the necessity for tracheotomy or intubation of the larynx is often averted.

All this is really ancient history, antitoxic serum having been first extensively used in 1894-1895, and there are not many alterations to record in the hospital treatment of diphtheria since this date. The history of the contest between the operations of tracheotomy and intubation of the larynx is, however, interesting. Before the days of antitoxin, tracheotomy was practically the only operation performed for the relief of laryngeal obstruction. Intubation was tried in 1892, but the results compared so unfavourably with those of tracheotomy that it was given up. Subsequently, however, and largely owing to the work of O'Dwyer and his followers in America, who effected considerable improvements in the apparatus, it was revived, and in some hospitals—at Edinburgh, for instance—it has practically replaced tracheotomy for the average case.

In considering this point, we must, however, first define what we mean by tracheotomy. This operation may be performed in two very different ways. In the older method a general anæsthetic was given, and a comparatively large incision was made in the neck, and the trachea was exposed by dissection, and the advice (given in most of the text-books of surgery) not to open the trachea till it could be seen was usually followed; often, quite a small tube was then inserted. Subsequently feathers were used to clear the tube of mucus whilst *in situ*. If this be taken as the standard method, there can be no doubt that intubation of the larynx is preferable. The use of chloroform adds enormously to the immediate risk of the operation, many patients having died on the table before the lengthy procedure for opening the trachea could be carried out; while the subsequent use of feathers (which could not be kept aseptic) has been shown to be often responsible for the supervention of broncho-pneumonia.

If, however, the trachea be opened by the sense of touch only, through a comparatively small incision, which does not open up the fascial planes of the neck, under a local anæsthetic (if necessary) only, and a large tube be inserted which does not require to be cleared with feathers, the results are very much better, so that in comparing intubation with tracheotomy it is very important to know which method of performing the latter is intended.

### Tracheotomy or Intubation?

The latter method has some marked advantages over intubation. It makes the whole calibre of the trachea at once available for respiration instead of the much smaller lumen of the intubation canula; it is possible to extract loose membrane from the trachea at the time of the operation instead of leaving this to block possibly the laryngeal tube in the larynx, and there can be no doubt that the after-treatment of the case is infinitely easier for the nurse, who is powerless if the patient should happen to cough up the intubation tube. It is, moreover, much the easier operation for the average man, though in the hands of an expert, intubation can be performed with great celerity.

At the present time tracheotomy is the operation of choice at most fever hospitals, though some medical officers who have become specially proficient with intubation prefer the latter procedure.

It was formerly the custom to place children before and after tracheotomy in steam tents, that is to say, in cots surrounded by curtains through which the nozzle of a steam kettle was allowed to protrude, thus enveloping the patient in an atmosphere of steam. This method had several disadvantages, one of which was that the clothes and bed linen soon became saturated with moisture, and the child was rendered liable to an attack of broncho-pneumonia. Another point was that the child being completely enveloped in curtains could neither be seen nor heard by the nurse on duty. When there were several of these tents in a ward it was necessary for the nurse to make a continual promenade from one to another in order to ascertain the condition of the child's breathing.

As a matter of fact a considerable improvement in the results followed the discontinuing of tents altogether, and at Monsall, for instance, I never used them at all, as I was convinced that they did more harm than good.

Latterly, however, in hospitals where a supply of steam exists in the ward for sterilising purposes it has been found useful to have a small jet playing into the air near the cot in the case of children suffering from laryngeal obstruction either before or after operation has been performed. The patient is not enclosed in any way, and can be continually watched by the nurse through an observation window in the duty room. At the Leeds City Hospital there is a small operating theatre opening into the special ward which is used for tracheotomy cases; the illustration shows this and the bedside steam apparatus. At the Edinburgh City Hospital steam is laid on to the general diphtheria ward, and the illustration shows two patients who have been intubated and are being treated in this way. By this means it is possible to obtain the advantages of a slightly warmed and moistened atmosphere without the risks of the steam tent.

We are fortunately on firmer ground with regard to the discharge of diphtheria patients than we are with scarlet fever, in that it is possible to recognise the organism of the disease by bacteriological examination. In every modern hospital nowadays no patient who has been suffering from diphtheria is allowed to leave the building unless his throat and nose have been examined with two or three consecutive negative results for diphtheria bacilli. Consequently, return cases of diphtheria are almost unknown, though before bacteriological examination was in vogue they used to be of fairly common occurrence. Similarly, every patient admitted with scarlet fever is examined for the presence of diphtheria bacilli in his throat or nose on his admission to hospital; if these are found he is treated in an isolation or separation ward. It is now well recognised that a child may have diphtheria bacilli in his mouth or nasal passages without obviously suffering from clinical diphtheria, but that in any given case we cannot be sure that he is not capable of infecting others with true diphtheria. There are bacilli which resemble those of true diphtheria which are not capable of producing this disease in others, but, inasmuch as they cannot be distinguished from true diphtheria bacilli without prolonged bacteriological examination, and sometimes without inoculation into susceptible animals also, patients harbouring anything resembling diphtheria bacilli in their throat or nose are treated as potentially infective to others and isolated accordingly. Personally, I found it quite safe to "barrier" these in the general ward for scarlet fever, and this procedure relieved what would have otherwise been a heavy strain on the isolation wards.

It seems almost impossible nowadays to imagine that only so far back as fifteen years ago diphtheria occurring in a patient who had been admitted for scarlet fever was regarded as a "complication" of that disease, but so it was.

Reverting for a moment to the treatment of diphtheria itself, while it is true that the great diminution in the mortality of this disease has been due to antitoxin, there is yet one other point worth mentioning—namely, that we have also learnt to leave the patients alone a little more. Formerly it was the custom to rely on vigorous treatment of the throat with various antiseptic applications which have now been shown to be not only useless but harmful, and have been therefore practically discontinued in fever hospitals generally. In private practice, however, there still remains some faith in local treatment, and there can be no doubt that this is often responsible for the omission to administer antitoxin in the early stage which still exists, and, as the statistics show, is so much to be regretted. Any vigorous treatment of the throat tends to weaken the patient which in a disease like diphtheria, with its tendency to attack the heart, is certainly to be avoided.

For the same reason the custom of employing a special nurse for each case after tracheotomy has been largely discontinued, as it was found that this often led to a good deal of over-nursing, or, rather, well-meant "fussing."

## Figures and Tables

**Fig. 1. f1:**
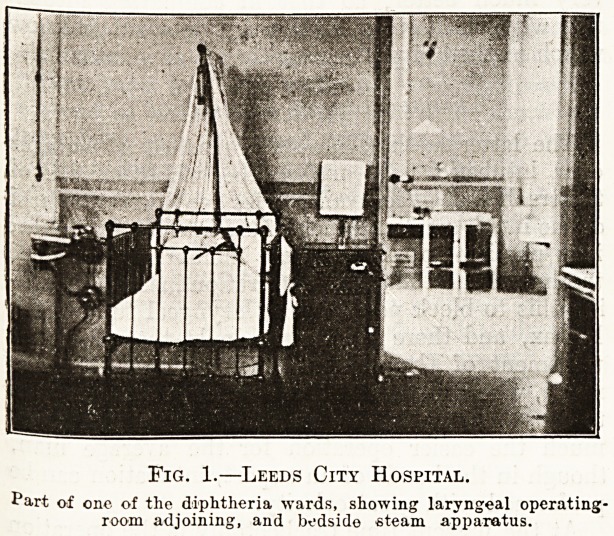


**Fig. 2. f2:**